# Supplementary material for: A scoping review of digital workplace wellness interventions in low- and middle-income countries
Source: PLoS One. 2023 Feb 28;18(2):e0282118. doi: 10.1371/journal.pone.0282118 (PMC9974126; doi:10.1371/journal.pone.0282118)
Supplement: S3 Table — (DOCX) [file pone.0282118.s003.docx]

**S3 Table. Supplementary table of included studies grouped by main targeted health outcome.**

1. **Lifestyle/ Chronic disease risk (n=7)**

| **Author, Year** | **Study settings** | **Design /Duration** | **Population characteristics** | **Recruiting inclusion criteria** | **Mode of delivery / Underlying theory**  **IG:** Intervention Group  **CG:** Control or Comparison Group | **Intervention providers** | **Health behaviour targeted** | **Outcomes and measures** | **Result (Qual & Quant)** | **Main findings** |
| --- | --- | --- | --- | --- | --- | --- | --- | --- | --- | --- |
| Jorvand (2020) | Iran  Healthcare workers in two healthcare networks with >120km distance from each other in Ilam province | Quasi-experimental study  2-week intervention with 6-month follow-up | **Baseline**  **Total (n)=**114  **Mean age=** 37.61±4.88 years  **Gender=** 50% males  **Education=** 58.77% with BSc degree  **IG**  **Total (n)=**59  **Gender=** 50.85% males  **CG**  **Total (n)=** 55  **Gender=** 50.9% females | **Inclusion criteria:**   1. Having literacy 2. Being formal or contractual employees 3. Not having daily exercise 4. Having access to the internet 5. Using social networks for education 6. Completed informed consent form   **Exclusion criteria:**   1. Resignation, dismissal, and failure to attend educational programs 2. Developing a disease or condition limiting the movement. | **IG:** Workers from one network received a Telegram-based intervention and supervised exercises. A Telegram group was created for:   - Sending **education packages** designed based on previous descriptive study results by the research group, every 2 weeks, providing thinking opportunities and reminding. - **Discussion and interlocution** - Participants could send their own exercise pictures. - Receiving **reminding messaging** to encourage doing exercise during the follow up phase.   **CG:** workers from the other network received uncontrolled individual exercise and self-reported.  **Underlying theory:** Health belief model | Research team | Effect on exercise as CVD preventive factor | **Primary outcome:**   1. **HBM** (perceived susceptibility, perceived severity, perceived benefits, perceived barrier and self-efficacy) 2. **Exercise** (daily and weekly exercise in minutes)   **Secondary outcome:** blood biochemical markers (FBS, TG, cholesterol, HDL and LDL) | **Significant difference of HBM constructs mean score at pre and post intervention in IG:**   1. **Increased perceived severity (p=0.000)** 2. **Increased perceived benefits (p=0.010)** 3. **Decreased perceived susceptibility (p=0.018):** directly related to increased preventive behaviours of doing exercise for CVDs 4. **Increased self-efficacy (p=0.024)**   **Significant effect of exercise in IG:**   1. **Daily exercise at post intervention (p=0.001)**: Interaction between time and group (IG =1.72±25.23 mins, CG= 4.16±1.09 mins) 2. **Weekly exercise at pre and post intervention (p=0.001)**   **Significant effect of mean blood indices at pre and post intervention in IG:** FBS (p<0.005)  **Significant difference between time and two groups at post intervention:**   1. Perceived susceptibility (p=0.036) 2. FBS (p=0.003) | Implementing educational interventions based-on Telegram messenger with emphasis on health belief and using HBM can improve exercise level. Thus, this application can be a suitable tool to deliver training when holding in-person is difficult. |
| Joseph-Shehu (2019) | Nigeria  University staff in Nigeria | Mixed concurrent design (Pilot study) with follow-up at 12-week | **Stage 1 for problem identification**  **Total (n)=** 340  **Quantitative (n)=** 280  **Qualitative (n)=** 24  **Poor PA=** 71.7%  **Poor health responsibility=** 73.9%  **Moderate stress management**= 60.4%  **Moderate nutrition=** 77.8%  **Moderate Interpersonal relation lifestyle practice=** 77.8%  **Baseline**  **Invited (n)=** 1349  **Included (n)=** 42  **Downloaded app (n)=** 25  **Age=** 38.4 ± 6.69 years (min=28, max=53)  **Gender=** 59.1% males  **Marital status=** 95.5% married  **Education=** 63.6% with first degree  **Monthly Income=** 50.0% with N101 000- N150 000  **Follow-up**  Completed survey (n)= 22 | **Inclusion criteria:**   1. University staff | **IG:** Adopted a nurse-client interactive Android phone app, Tertiary Staff Health Promotion App (TASHePA) which consisted of both worker and nurse interfaces to access health promotion information, to monitor health status, increase PA and minimize sitting hours, with reminder of activities that will improve health and quality of life.   - ***Contact Us* page:** For interaction purpose between participants and nurses - ***Living Healthy* page:** Provides in-built tips on HPLB relevant on a daily basis - ***Step Tracking* page:** encourage PA, preventing overweight and obesity - ***Alarm* page:** daily in-built activity enhancing individual HPLB and health status - ***Health Examination* page:** track participants’ health status including blood pressure, weight, WHR and FBG   **Underlying theory:** Health promotion model  **No CG applied** | Research team | Health promoting lifestyle behaviour (HPLB) | **Primary outcome:** HPLB and health status (BP, BMI, WHR, and FBS) | **Significant difference between pre- and post-intervention:**   1. Increased nutrition score (p=0.0001): +2.77±0.69 2. Increased PA score (p=0.0001): +2.50 ± 0.51 3. Increased health responsibility score (p=0.0001): +2.73 ± 0.63 4. Increased stress management score (p=0.001): +2.95 ± 0.65 5. Interpersonal relation subscales score (p=0.009): +3.00 ± 0.69 6. Decreased BMI (p=0.038): -27.32 ± 5.34 kg/m2 7. Decreased diastolic BP (p=0.04): -72.95 ± 11.74 mm Hg | The health-promoting lifestyle behaviour and health status of workers and other population groups showed improvements through information and communication technology. |
| Liu (2015) | China  Staff at the health management centre of a hospital from eight units | Clustered RCT  12-month intervention | **Voluntary**  **Baseline**  **Recruited (n)=** 589  **Completed 12-month follow-up (n)**= 427 (72.5%)  **Average age=** 60.57 years  **Gender=** 58.2% female  **Education:** 56.2% college or above  **Income:** 43.1% have ≥RM5k/month  **IG**  **Baseline (n)=** 238  **Follow-up (n)=** 163  **Mean age**= 58.72±8.92 years  **Gender**= 58.4% males  **Education**= 54.2% college or above  **CG**  **Baseline (n)=** 351  **Follow-up (n)=** 264  **Mean age**= 61.82±8.8 years  **Gender**= 58.1% males  **Education**= 51.6% college or above | **Inclusion criteria:**   1. Have been allocated to have a medical examination at the hospital for more than 2 years at the baseline 2. Without known CVD 3. Aged 45-75 years   **Exclusion criteria:**   1. Had mental health abnormalities history 2. Difficulty in communication, such as reading or answering the questionnaire 3. Unable to understand the aim of the study 4. Currently participating in another clinical trial or had done within the previous 6 months | **IG: mobile phone-based lifestyle intervention program**   - received an individualised electronic prescription and a handbook for CVD health education derived from the Chinese guideline for CVD prevention and PREMIER trial (a successful intensive lifestyle intervention) within 2 weeks after baseline medical examination. The health content includes demonstrations of healthy dietary patterns and cooking methods, PA, weight management, smoking and alcohol reduction, and healthy psychological conditions. - Received follow-up 5 to 8 min phone calls and text messages targeting reducing CVD risk during the 12-month intervention. The frequency based on the participants’ CVD risk level.   **CG:** receive usual medical examination without follow-up calls and text messages.  **No theory applied** | Research team | **Cardiovascular Disease (CVD) risk** | **Primary outcome:**   1. **Change in 10-year CVD risk:** measured via well-validated assessment methods for CVD predictions from age, Systolic Blood Pressure (SBP), BMI, Total Cholesterol (TC), diabetes and smoking, ischemic CVD.      1. **Change in components of risk score:** SBP, TC, BMI   **Secondary outcome:**   1. **Diastolic blood pressure (DBP)**: measured with an automatic Omron sphygmomanometer in sitting position after 5 min of rest 2. **Triglyceride (TG)**: determined by automated enzymatic methods 3. **HDL and LDL cholesterol:** tested by homogeneous method 4. **Fasting plasma glucose (FPG)**: measured by hexokinase enzymatic method 5. **Waist-to-hip Ratio (WHR)** | **Significant difference between baseline and 12-months in CG:**   1. Increased mean CVD risk: +1.77% 2. Increased SBP: +6.89mmHg (p<0.001) 3. Increased DBP: +5.62mmHg (p< 0.001) 4. Decreased HDL and LDL: (p<0.05) 5. Deceased overall DBP (-12.23mmHg), FPG (FPG: -0.32mmol/L) and WHR (-0.02) (p<0.001)   **Significant difference between baseline and 12-months in IG:**   1. Decreased 10-year CVD risk (SBP, TC, BMI) (p<0.05) 2. Decreased DBP: -6.61mmHg (p<0.001) 3. Decreased HDL and LDL (p<0.05)   **Significant difference between IG and CG:**   1. Decreased DBP in IG: -12.23mmHg (p<0.001) 2. Decreased FPG in IG: -0.32mmol (p<0.001) 3. Decreased WHR in IG: -0.02 (p<0.001) | Mobile phone-based intervention may therefore be a potential solution for reducing CVD risk in China. |
| Martinez (2018) | Latin America (Bolivia, Guatemala, Paraguay)  Hospital workers from three organisations | Pre-post study (evaluate before and 6 months after training)  6-month intervention with follow-up | **Voluntary**  **Baseline**  **Included (n)=** 202 with 54.0% doctors  **Gender=** 72.28% females  **Age (n)=** 50.99% ≤35 years  **Countries**  **Bolivia (n)=** 76  **Guatemala (n)=** 79  **Paraguay (n)=** 47  **Smoker**  **Current (n)=** 24  **Former (n)=** 29  **None (n)=** 149  **Complete pre-post evaluation (n)**=99 | **Inclusion criteria:**   1. Being a hospital worker 2. Have an email account   **Participants characteristic:**   1. Participant hospital had previously collaborated with the Trainings Unit of the ICO in training their health professionals in either e-learning or in-person course | **IG:** received an adapted version of a 5A’s (Ask, Advise, Assess, Assist and Arrange follow-up) training program developed by the online platform e-oncologia based on in-person courses.  The online training program is composed of 4 modules and includes slides, review exercises, practical cases and practical solving exercises. The course has been accredited by the Council of Oncology in Europe.  **No CG and theory applied** | Local coordinator recruited the participants over 6 months and reminders message to attend post-training evaluation | Smoking cessation | **Primary outcome:** Cognitive and behavioural factors relating to smoking by assessing the individual, behavioural, and organisational-level factors through a questionnaire that included 43-item (0 = none, 10 = most possible) completed before and 6 months after the training.  **Secondary outcome:** Self-reported performance level according to 5A’s, demographics characteristic and questions suggested by experts to explore behavioural factors | **Significant Increase in performance of each of the 5A components (p<0.001):** Ask (7 to 9), Advise (7 to 9), Assess (6 to 8), Assist (2 to 7), Arrange a follow up (0.52 to 5)  **Significant improvement at post training**   1. **Performance score of 5A’s (p<0.001):**  - Doctors > others - Former smokers > smokers and non-smoker - Participants without previous training - Countries: Paraguay > Bolivia and Guatemala at both pre and post training  1. **Cognitive, behavioural and organisational factors affecting 5A’s**  - **Five identified barriers (p<0.001):** self-reported preparedness, drug preparedness, competency in assisting smokers to quit, using additional resources and having positive experience. - **Opportunities with score ≥7 (p<0.001**): motivation to help patients to quit, importance of smoking cessation in job, seeking frequently for patients. | The online training had a positive impact on the implementation of the brief intervention. Online education on smoking cessation is feasible and effective in improving smoking cessation interventions in these countries. |
| Nurgul (2015) | Turkey  Female staff from Sakarya University | Descriptive and quasi experimental study  3-month intervention without follow-up | **Voluntary**  **Baseline**  **Total (n)=** 44  **Included (n)=** 30  **Age**= 26-32years (56.7%)  **Professional experience**= 1-5 years (50%)  **Marital status=** Married (53.3%) | **Inclusion criteria:**   1. Working at the university 2. Able to use computer 3. Own a computer connected to Internet either at home or at work 4. Accept to participate in the research 5. Had filled in the form related to research and completed the training | **IG:** Web-supported health education training material in ppt or audio-visual provided for 3 months. The training material consists of 3 modules: nutrition and health, physical activity, damages of smoking and stress management, available 7 days a week and 24 hours a day.  **No CG and theory applied.** | Research team | **Knowledge on health promotion** | **Primary outcome:**   1. **Knowledge on health promotion:** assessed by multiple choice questionnaire 2. **Individual’s health behaviours:** assessed via 52-items health promotion lifestyle profile (HPLSP) with 6 sub-dimensions (Health Responsibility, PA, Nutrition, Self-actualization, Interpersonal support, stress management) | **Significant difference (p<0.05) between pre and post intervention**   1. HPLSP total points (p=0.001) 2. HPLSP sub-scale total points (p=0.001) 3. MCQ (p<0.001) | Web-based health education had a positive effect on healthy lifestyle behaviours of women staff working at Sakarya University and on their knowledge of health protection. |
| Ramachandran (2013) | India  Male employees in public and private sector industrial units in southeast India (Chennai, Tamil Nadu, Visakhapatnam, Andhra Pradesh) | Prospective and parallel RCT  24 months intervention with follow-up | **Voluntary**  **Baseline**  **Total (n)=** 2744  **Included (n)=** 537 (19.5%)  **IG**  **Total (n)=** 271  **Mean age=** 45.9 ±4.8 years  **BMI=** 25.8 ±3.3 kg/m^2^  **CG**  **Total (n)=** 266  **Mean age=** 46.1 ±4.6 years  **BMI=** 25.8 ±3.0 kg/m^2^ | I**nclusion criteria:**   1. No diabetes (self-reported) or major illness such as cancer, chronic liver or kidney disease, disorders with cognitive impairment, severe depression or mental imbalance 2. No physical disability that would prevent regular PA 3. No recruitment in another trial      1. Aged 35–55 years and BMI ≥ 23 kg/m² 2. Own a mobile phone, able to read and understand mobile phone messages in English 3. Positive family history of type 2 diabetes (T2D) | **IG**= Frequent mobile phone messages contained information about healthy lifestyle, the benefits of PA and diet, cues to start PA and healthy dietary practices, and strategies to avoid relapse and remain motivated to maintain PA and healthy dietary habits.  **CG**= standard lifestyle modification advice at baseline only  **Underlying theory:** Transtheoretical Model of Behavioural Change | Research team  Mobile phone messaging website was created by the researchers in partnership with Intel | PA, diet | **Primary outcome:** Incident of T2D.  **Secondary outcomes:** BMI, waist circumference, systolic and diastolic blood pressure, lipid profile (total cholesterol, HDL and triglycerides), total dietary energy intake, PA score. The acceptability of mobile phone messaging was assessed by questionnaire in the IG. | **Develop T2D over two years:** 50 (18%) IG participants V.S. 73 CG participants (27%) (absolute risk reduction 9%; 2).  The intervention reduced the incidence of T2D during the course of the study (β –0·447).  The number needed to treat to prevent one case of T2D was 11 (95% CI 6–55). | Mobile phone messaging is well-accepted and effective for lifestyle modification to reduce incidence of T2D. |
| Rubinstein (2016) | Latin America  Adult men and women with prehypertension from health care centres, workplaces and community centres from Argentina, Guatemala, Peru | Parallel RCT  12-month intervention with 6-month follow-up | **Voluntary**  **Baseline**  **Total (n)=** 2630  **Eligible (n)=** 1993  **Included (n)=** 637  **IG**  **Total (n)=** 316  **Completed (n)**= 26  **Mean age=** 43.6±8.4 years  **Gender=** 53% females  **Marital status=** 73% married  **Received introductory calls (n)=** 18 (7%)  **Counselling calls**  **Received 50% (n)=** 188 (69%)  **Received >75% (n)=**111 (41%)  **Received 100% (n)=**7 (3%)  **Received text message (n)**= 13-32, with max 48  **CG**  **Total (n)=** 321  **Completed (n)**= 287  **Mean age=** 43.2± 8.4 years  **Gender=** 54% females  **Marital status=** 72% married | **Inclusion criteria:**   1. Aged 30-60 years had SBP of 120-139mmHg, DBP of 80-89 mmHG or both (confirmed by research assistant) 2. Own mobile phone for personal use 3. Able to read and understand text messages in Spanish   **Exclusion criteria:**   1. Taking antihypertensive drugs 2. Pregnant women 3. People who are illiterate 4. People with previous diagnosis or treatment of hypertension, diabetes or CDV | **IG:** Received monthly scheduled 20-30mins motivational counselling calls and weekly personalised text messages to their mobile phones about diet quality and PA. The text messages serve as reinforcement after counselling calls (numbers correspond to calls).  **CG:** Received usual care  **Underlying theory:** Transtheoretical Model of Behavioural Change and the Health Belief Model | Research team | Lifestyle improvement (blood pressure, weight loss, diet quality and PA) | **Primary outcome:**   1. Change in SBP (mmHg) 2. Change in DBP (mmHg**)**   **Secondary outcome:**   1. Change in weight (kg) 2. BMI (kg/m2) 3. Change in waist circumferences, WC (cm) 4. Change in PA (METS/ min per week) 5. Change in daily intake of F&V 6. Change in daily intake of high fat and high sugar foods | **Significant changes in highly engaged participants (received ≥75% of counselling calls in IG):**   1. **Body weight:** -4∙85kg 2. **WC:** -3∙31cm 3. **Daily intake of F&V:** +0.66 4. **Daily intake of high-sodium foods:** -0.42 5. **Daily intake of high fats food:** -1.52   **Significant differences in IG:**   1. **Body weight (p=0·04):** –0·66 kg 2. **BMI (p=0.02):** -0.30 3. **Daily F&V intake (p=0.05):** +0.25 4. **Daily intakes of high fat & sugar food (p=.008):** -0.75 5. **WC in men:** -1.35cm 6. **BMI in participants >45years:** -0.33   **Significant difference between IG and CG by country:**   1. **Body weight in Peru (IG):** -1.24kg 2. **Daily F&V intake in Peru (IG):** +0.64 3. **Daily F&V intake in Guatemala (IG):** +0.04 | The mHealth-based intervention did not result in a change in BP that differed from usual care but was associated with a small reduction in bodyweight and an improvement in some dietary habits. A dose-response effect was seen which signals potential opportunities for larger effects from similar interventions in low-resource settings. |

1. **Weight management (n=5)**

| **Author, Year** | **Study settings** | **Design /Duration** | **Population characteristics** | **Recruiting inclusion criteria** | **Mode of delivery / Underlying theory**  **IG:** Intervention Group  **CG:** Control or Comparison Group | **Intervention providers** | **Health behaviour targeted** | **Outcomes and measures** | **Result (Qual & Quant)** | **Main findings** |
| --- | --- | --- | --- | --- | --- | --- | --- | --- | --- | --- |
| Abdi (2015) | Iran  Employees, chairmen and managers from Governmental offices, organizations and institutions | Three-Arm RCT  6-month intervention with 3-month follow-up and maintenance | Voluntary  **Baseline**  **Total (n)**= 435  **Follow-up (n)**= 401  **Mean age**= 42 years  **Gender**= 71.3% females  **Education**= Bachelor's degree and higher  **Working experience**= 16-18 years | **Inclusion criteria:**   1. BMI >25 kg/m^2^ 2. Working for at least 8hrs/week 3. Having computer and to be familiar with working with computer 4. Able to access, being familiar with the telephone, cell phone and the Internet 5. Age above 18 years.   **Exclusion criteria:**   1. Pregnancy 2. Depression and other disorders leading to PA | **IG: receive lifestyle program and receive general brochures:**   1. **Web-assisted group**: Educational content about healthy nutrition and PA skills provided on a website. 2. **Telephone assisted group**: Same educational content sent through SMS every two weeks   Telephone and email consultations were provided every two weeks for both intervention groups  **CG:** only receive general brochures about lifestyle and overweight  **Underlying theory:** Social cognitive theory (SCT) | 1. Educational content and consultations delivered by the research team 2. There are respected participants served as a role models (observational learning) | Weight control of the employees with overweight and obesity | **Anthropometric measures:**   1. **Weight**: measured by digital scale (Trillion), a standard 5kg weight (as control) was used after each 10 times of weight measurement 2. **Waist circumference**: measured by measuring tape twice midway between the lowest rib and the iliac crest. 3. **Blood pressure**: measured with aneroid sphygmomanometer   **SCT measures:** Researcher-made questionnaire based on the Dishman and Dewar questionnaire (including scales of self-efficacy, intention, situation, social support, behavioural strategy, outcome expectations and expectancies), completed twice by 20 employees. | The lifestyle intervention resulted in a weight loss:   1. 1.92kg in telephone-assisted group 2. 1.08 kg web-assisted group   **Telephone-assisted group**: mean scores of the constructs of self-efficacy (P=0.001), environment (P=0.001), outcome expectations (P=0.040), and outcome expectancies (P=0.001) increased.  **Web-assisted group**: mean scores of the constructs of self-efficacy (P=0.001) and outcome expectancies (P=0.020) increased.  A significant difference (P=0.03) was observed in weight loss among the groups in the course of time. | Results showed the effectiveness of the intervention based on new communication technologies and the Social-Cognitive Theory.  Future studies with more retention strategies regarding self-efficacy and environment constructs are needed to further explain the application of SCT and technology-based approaches to reduce obese and overweight. |
| Beleigoli (2020) | Brazil  Overweight and obese adults  students and staff of a Brazilian university | Three-arm parallel RCT  24-week intervention | **Voluntary**  **Baseline**  **Total (n)=** 3745  **Included (n)=** 1298  **IG (Platform only)**  **Total (n)=420**  **Mean age=** 34.4 years  **Weight=** 83.4 kg  **BMI=** 30.12 kg/m^2^  **Gender=** 75% females  **IG (Platform and coaching)**  **Total (n)=408**  **Mean ag =** 33.0 years  **Weight=** 82.3kg  **BMI=** 29.85 kg/m^2^  **Gender=** 78.2% females  **CG**  **Total (n)=470**  **Mean age=** 33.4 years  **Weight =** 82.6kg  **BMI=** 29.73 kg/m^2^  **Gender=** 77% females | **Inclusion criteria**:   1. Age 18-60 2. BMI≥ 25/kgm2 3. Intention to lose weight through a behaviour change program 4. Having web access   **Exclusion criteria:**   1. Pregnancy 2. With specific dietary requirements 3. Participation in other weight loss programs | **Platform-only group (IG1):** received reminder emails to report weight and habits through the platform at 12 and 24 weeks after baseline trial and get access to 24-week weight loss program delivered through web platform with personalised computer-delivered feedback.  **Platform and coaching group (IG2):** received 24-week web-based weight loss program with 12-weeks personalized feedback delivered online by a dietitian  **CG (wailist):** received a non-personalized minimal intervention based on dietary and PA recommendations delivered through downloadable e-booklet and four 5-min videos.  **Underlying theory:** Behaviour Change Wheel | Research team | Weight loss | **Primary outcome:**   1. **Self-reported weight at 24-week:** encouraged through messages on the web platform to continuously report 2. **BMI changes**   **Secondary outcome:**   1. **Changes in dietary:** assessed by the daily F&V portions, weekly consumption of sweetened beverages and ultra-processed foods through the Brazilian food frequency questionnaire (Questionário de frequência alimentar] and reported over the web platform after 24-week 2. **Changes in PA**: Moderate and vigorous PA was assessed by the Brief PA Assessment Questionnaire. | **After 24 weeks intervention,**   1. **Primary outcome (Weight & BMI change)**  - **CG:** Weight= -0.66kg, BMI= -0.24kg - **IG1:** Weight= -1.08kg, BMI= -0.38kg - **IG2:** Weight= -1.57kg, BMI= -0.56kg - **Significant overall weight loss (p=0.001):** IG1 (83/420, 19.8%) and IG2 (64/408, 15.7%) > CG (61/270, 13.0%)  1. **Secondary outcome:**  - **Significant increase in F&V consumption (p=0.001):** IG1 and IG2 > CG - **Significant reduction in ultra-processed food consumption (p=0.005):** IG1 and IG2 > CG - **Significant increase in sweetened beverage consumption (p=0.02):** IG1> IG2 | The web-based behaviour changes programs with computer- and human-delivered personalised feedback led to greater, albeit small-magnitude, weight loss within 24 weeks. Improvement in multiple dietary habits, but not PA, were also greater in the personalised programs compared with the non-personalized one.  The human-delivered personalised feedback by the online dietitian coach increased user engagement with the program and was associated with a significantly higher chance of clinically meaningful weight loss. |
| He (2017) | China  Occupational population from 134 government agencies and enterprises | Cohort Study  6-month intervention with 6-month follow-up | **Voluntary**  **Baseline**  **Total (n)=** 15,818  **IG (n)=** 12,296  **CG (n)=** 3522  **Data collected**  **Total (n)**= 15,523  **IG (n)**= 12,002  **CG (n)**= 3521  **After 6-month intervention**  **Total (n)**= 15,310  **IG**  **Total (n)**= 11, 843 (77.35%)  **Gender**= 66.53% females  **Age**= 68.552% <40 years  **Education**= 91.85% high  **CG**  **Total (n)**= 3467 (22.65%  **Gender**= 59.53% males  **Age**= 50.61% <40 years  **Education**= 77.69% high) | **Inclusion criteria:**   1. From one of any of the 134 government agencies and enterprises in Shunyi District 2. Aged 18 years and above 3. Wanted to lose weight   **Exclusion criteria:**   1. Pregnant women and with other health conditions which were not suitable for weight loss | **An observational study only involved data collection at pre- and post-intervention.**  **IG: social media-based intervention** Participants willing to use the research team’s official WeChat account were enrolled in a WeChat group. WeChat account consisted of 6 components:   1. **Introduction** 2. **Weight loss process:** provide feedbacks on weight, diet and exercise to motivate participants to lose weight through weekly report 3. **Weight loss unit rankings:** rank total score per unit for competitive purpose 4. **Weight loss school:** Variety materials on weight loss, including micro videos and popular science knowledge 5. **Activity area:** micro community and other types of activities for communication purpose 6. **Rewards**: through cumulative score of various activities.   **WeChat:** WeChat message, micro community discussion based on the post, online consultation by experts  **CG**: Participants not willing to use official WeChat account given routine publicity such as slogan “take the stairs and lose weight” on weight loss  **No theory applied.** | Shunyi District Government | **Weight loss behaviour** | **Primary outcome:**   1. **Weight loss:** height, weight, waist circumferences before and after intervention collected 2. **Demographic characteristics**: gender, age, educational level and telephone number online registered with WeChat account | **Weight loss:**   1. **IG** (2.09±3.43kg) > **CG** (1.78±2.96kg) 2. Significant weight loss between males in IG and CG based on stratification of age and education level:  - Males aged <40 years and ≥40years with low education level has significant weight loss (p=0.001) - Males aged <40 years (p<0.001) and ≥40years (P=0.03) with low education level has significant weight loss   **Effect of WeChat on weight loss (Assessed with propensity method, p<0.05):**   1. Males in IG (active or inactive) had higher probability of maintaining weight with 1-2kg or > 2kg weight loss than CG (0-1kg) 2. Active participants in WeChat groups are more likely to lose weight. | The weight loss intervention campaign based on an official WeChat account focused on an occupation-based population in Shunyi District was more effective for males than females. |
| Limaye (2017) | India  Young Indian employee in 2 multinational the information technology industry in Pune | RCT  1-year intervention | **Voluntary**  **Baseline**  **Total (n)=** 437  **Included (n)=** 265 (60.6%)  **Mean age (years)=** 36.2 ± 8.0 years  **Gender=** 72.5% males  **IG**  **Total (n)=**133  **Drop-outs (n)**=28 (21.1%)  **CG**  **Total (n)=**132  **Drop-outs (n)**= 34 (25.8%) | **Inclusion criteria**:   1. Participants with ≥3 risk factors (family history of cardiometabolic disease, overweight/obesity, high BP, impaired FBG, hypertriglyceridemia, high LDL and low HDL)   **Exclusion criteria:**   1. With diabetes, hypertension or lipid abnormalities requiring treatment, major illness 2. Disability restricting PA 3. Pregnant women | **IG**: Receive information on lifestyle modification through mobile phone messages and emails. After a survey of participants’ preferences, 3 mobile phone messages and 2 e-mails with infographics were sent per week between 10am–1pm; no message was repeated. Additional support through a website (requires login) and a closed Facebook group.  **CG**: no intervention received  **Underlying theory:** goal setting | N/A | Diet, exercise, stress management, lifestyle, weight loss, adherence | **Primary outcome**: prevalence of overweight/obesity (BMI ≥ 25 kg/m2)  **Secondary outcome**: Change in weight, waist circumference, blood pressure, glucose, lipid, lifestyle choices (PA, frequencies of calorie-dense and fibre-rich foods, smoking), diabetes awareness score, acceptability and cost-effectiveness of the intervention | **Significant weight loss in IG (p<0.05):** Overweight/obese decreased from 104 (78.2%) to 96 (72.2%)  **Significant weight gain in CG (p<0.05):** Overweight/obese number increased from 101 (76.5%) to 110 (83.3%)  Risk difference= 11.2% (95% CI 1.2–21.1; P=0.042).  The number-needed-to-treat/prevent one case of overweight-obesity in 1 year was 9. | A virtual assistance-based lifestyle intervention was effective, cost-effective and acceptable in reducing risk factors for diabetes in young employees in the information technology industry and is potentially scalable. |
| Yu (2018) | China,  Employees from institutions or enterprises from four areas (Beijing, Guangdong, Henan, Shaanxi) | RCT  3-month intervention | **Voluntary**  **Baseline**  **Total (n)=** 904  **Included (n)=** 802  **Gender=** 50.49% females  **Overweight and obese (n)=** 360  **Normal weight (n)=** 398  **Underweight (n)=** 44  **Followed exercise program (n)=** 718/802 (89.5%)  **Completed exercise program (n)=** 688/802 (85.8%) | **Inclusion criteria:**  1. Full-time employees willing to manage their weight and improve their lifestyle  2. Aged 18–65 years.  **Exclusion criteria:**  1. Secondary obesity  2. History of weight-loss interventions (bariatric surgery, acupuncture, moxibustion, or prescribed medication), and significant changes (> 5%) in body weight in the past 3 months  3. Diagnosed osteoarthritis, abnormal physical deformities and history of spine or limb surgery or fracture in the past 3 months  4. Severe functional disorder or organic diseases of the heart, liver or kidney  5. Uncontrolled hypertension (Blood Pressure 180/110mmHg) or its complications  6. Fasting Blood Glucose (FBG) of 16.7mmol/L or diabetic complications  7. Pregnant or lactating women. | **IG:** Receive self-monitored intervention trial, involving individualised pedometer-assisted exercise prescription and a one-time targeted dietary guidance prior to exercise. Participants were also asked to synchronise exercise data of the pedometer to the Internet-based Health System Centre daily (at least weekly), by connecting to the personal computer (PC) using a USB cable or via Bluetooth  **No CG and theory applied** | Research team | Effectiveness of weight management program through PA and diet prescriptions | **Primary outcome:**   1. **Changes in body weight**: measured by BIA, accurate to 0.1kg 2. **BMI**: calculated by dividing the weight by the square of height (kg/m^2^) 3. **Waist circumference (WC)**: measured in accordance with the guidelines for prevention and control of overweight and obesity in Chinese adults, nearest to 0.1cm. 4. **Blood pressure (BP)**: measured with the right arm using mercury sphygmomanometer.   **Secondary outcome:**   1. **Changes in lifestyle behaviour**s: calculated by six aspects behaviour score include exercise, diet, smoking, smoking, drinking, stress handling and diseases prevention. 2. B**ody fat percentage (BF %)**: measured using BIA, accurate to 0.1% 3. **FBG**: measured using automatic blood biochemical analyser 4. **Serum lipid** (TG, total cholesterol, LDL-C, HDL-C): measured using automatic blood biochemical analyse   **Health assessment**: physical examination and blood chemistry testing, medical history and treatment, dietary questionnaire on lifestyle, data recorded in IHSC | **Overweight/obese participants:**   1. **Decreased weight**: -2.2% (BW/BMI) (p<0.001) 2. **Decreased WC: -**1.8% (p<0.001) 3. **Decreased %BF:** -3.3% (p<0.001) 4. **Decreased SBP:** -3.3mmHg (p<0.05) 5. **Decreased DBP:** -3.2mmHg (p<0.05) 6. **Decreased FSG:** -0.11mmol/L (p<0.05) 7. **Decreased TC:** -0.13mmol/L (p<0.05) 8. **Overall weight loss:** -68.2% (208/305), of the 68.2% with 3.5% reduction and 20.2% (42/308) achieve weight loss ≥ 5%   **Normal-weight individuals**   1. **Decreased weight:** -0.7% (BW/BMI) (p<0.05) 2. **Decreased %BF:** -2.5% (p<0.05) 3. **Decreased WC:** -0.8% (p<0.05) 4. **Decreased DBP:** -1.4% (p<0.05) 5. **Decreased FBG:** -2.4% (p<0.05) 6. Increased lifestyle behaviour score (p<0.001)   **Underweight participants**   1. Weight gain by 1.0%, with significant average increase of 0.5kg and 0.18kg/m^2^ BMI **(p<0.05)** 2. Lifestyle behaviour score improve **(p<0.001)** | The prescription pedometer-assisted walking intervention can effectively improve exercise adherence and manage weight. This approach was also effective in controlling the risk factors of weight-related chronic diseases |

1. **Physical activity (n=4)**

| **Author, Year** | **Study settings** | **Design /Duration** | **Population characteristics** | **Recruiting inclusion criteria** | **Mode of delivery / Underlying theory**  **IG:** Intervention Group  **CG:** Control or Comparison Group | **Intervention providers** | **Health behaviour targeted** | **Outcomes and measures** | **Result (Qual & Quant)** | **Main findings** |
| --- | --- | --- | --- | --- | --- | --- | --- | --- | --- | --- |
| Blake  (2019) | China  Employees of IT private sector organisations in Beijing and Guangzhou | Two arm cluster RCT  12-week intervention | **Baseline**  **Total (n)=** 690  **Included (n)**= 282  **Age**= 25-40 years  **Job type=** 75% computer programmers and project managers involves in conferences call with clients  **IG**  **Total (n)**=490  **Completed baseline survey and included (n)=** 196  **Gender=** 50.5% males  **Education=** 87.5% University  **Marital status=** 60.2% Married  **CG**  **Total (n)=**200  **Completed baseline survey and included (n)=** 86  **Gender=** 57.0% males  **Education=** 95.3% University  **Marital status=** 72.1% Married | **Inclusion criteria:**   1. Employees of the organisation | **IG (Guangzhou):** “Move-It” digital video-based worksite exercise intervention   1. **Move-It website**: 6 video clips series demonstrating Qigong exercises designed to be undertaken twice/day for 10 mins every working day, a set exercise break times were posted in sequence, one every 2 weeks on the webpage 2. **Reminder messages:** The Move-it icon on employees’ desktop computer screen popped up twice/day, at the same time every day. 3. **Exercise adherence info**: Individual daily exercise logs collected when clicked on the Move-it screen icon   **CG (Beijing, wait-list)**: receive the same intervention after the intervention  **Underlying theory:** Behaviour Change Techniques (BCTs) | 1. Research team 2. Company team leaders (n)=31 act as intervention facilitators to deliver intervention and encourage co-workers to engage | PA and work performance at the workplace | **Primary outcome:**   1. **PA**  - **PA level:** assessed by IPAQ, Taiwanese short form via self-reporting, questions including exercise frequency and duration and calculated through score. - **Weekday sitting hours:** assessed by score through number of sitting hours at work, home and travel - **Exercise adherence:** daily exercise frequency collected through interactive computer-based system while Qigong-related adverse events were reported by team leaders to the research team  1. **Work performance:** assessed by WHO and Work Performance Questionnaire, measuring on a 10-point scale.   **Questionnaires:** Chinese and English version were provided through online | **PA outcome:** Significant increased PA hours/ week in IG (5.80 h/w, p=0.04) and CG (7.41 h/w, p=0.00) but non-significant difference in the changes between groups (p = 0.70)  **Work performance:** Significant increase in CG (+ average 0.69 units, p=0.01) than in IG (− average0.03 units, p=0.78)  **Sitting hours:** Significant increase in IG (10.34 h/w, p=0.00) and CG (5.68 h/w, p=0.00), with significant difference in changes between group (−4.66 h/w, p<0.01)  The intervention did not result in greater changes in the intervention group than in controls.  Many participants perceived the Qigong exercises positively and reported positive benefits on physical and mental health including muscle relaxation, stress reduction and improved working mood | Delivery of a digital Qigong worksite exercise intervention was successful in raising awareness of the importance of PA and had wide reach and good uptake.  The intervention showed to be feasible and was acceptable to both managers and employees. long-term commitment of the organisation to promoting exercise at work remains unknown. |
| Ganesan (2016) | Asia, Europe, Africa, North America, South America, Australia, and New Zealand,  Adult employees from private and public sector organisations | Prospective cohort study  100-day intervention annual program  **No follow up** | **Voluntary**  **Baseline**  **Total (n)=** 69,219  **Non-completed Stepathlon=** 32, 657 (47%)  **Completed Stepathlon=** 26,562 (53%)  **Mean age=** 36 years  **Gender=** 76.2% males  **Countries (n)= 6**4, 92% LMICs with largest participants include India (90.2%), Australia (5%), New Zealand (1.1%), Singapore (0.6%)  **Indian participants (n)=** 26,562 (90.2% out of total)  **Weight pre-Stepathlon=** Overall (73.6± 13.3 kg), Males (76.6± 12.1 kg), Females (64.6±12.8 kg) | **Inclusion criteria:**   1. Adult employees from private and public sector organisations 2. Completion of baseline data collection | **A collaborative project with Flinders university and University of Adelaide. The current study only involved data collection via web-based questionnaires before and after the 100-day program.**  **IG: 100-day** Stepathlon workplace-based pedometer program   1. **Pedometer** (3D piezoelectric accelerometer technology) to record step count in an interactive challenge among participants. 2. **Stepathlon website:** facilitate motivation and engagement of participant  - Downloadable mobile device app and available on the internet - Provide self-monitoring personalised tools including logs of exercise and diet intake - Educational content with papers on nutrition and lifestyle - Platform for the user to post comments and also chat with research team  1. **Daily email**  - Deliver educational and encouragement messages to trigger individual and team milestones - **Entertaining:** simple quizzes and competitions to encourage online interface interaction   **No CG and theory applied** | Researchers at Flinder university and university of Adelaide and Stepahlon private limited in Mumbai, India | Effect of Stepathlon on PA | **Primary outcome:** PA level (step count, sitting duration, exercise duration, weight loss) through web-based survey questionnaires at pre- (1 week prior) and post- (1-2 week) intervention. | **Post program showed significant improvements in**:   1. Recorded **step count** (+3,519 steps/day, p<0.0001) 2. **Exercise days** (+0.89days, p<0.0001) 3. **Sitting duration** (-0.74hrs, p<0.0001) 4. **Weight** (-1.4 kg, p<0.0001)   Improvements occurred in women and men, in all geographic regions, and in both high and low-middle income countries, and the results were reproduced in 2012, 2013, and 2014 cohorts.  **Predictors of weight loss all (p < 0.0001)**:   1. Step= increase 2. Sitting duration= decrease 3. Exercise days= increase | Distributed mHealth implementation of a low-cost life-style intervention is associated with short-term, reproducible, large-scale improvements in PA, sitting, and weight. |
| Gu (2020) | China  Workers from 17 worksite | Quasi-experiment with self-controlled design  100-day intervention with follow-up | **Baseline**  **Total (n)=** 398  **IG (n)=** 221  **CG (n)=** 177  **Follow-up**  **Total (n)=** 262 with 68.3% adherence  **IG (n)=** 150  **CG (n)=** 112  **IG**  **Total (n)=150**  **Age=** 37.4% with 30-39 years  **Gender=** 60% males  **Marital status=** 76.7% married  **CG**  **Total (n)=112**  **Age=** 38.4% with 30-39 years  **Gender=** 50% males  **Marital status=** 61.6% married | **Inclusion criteria:** no willingness to resign within a year  **Exclusion criteria:** presence of heart disease, cerebrovascular disease, mental illness, or physical disorders. | **IG:** a pedometer- and group-based intervention program which consisted of 47 groups with 10-20 participants each. Each group chose one person as group captain.  **Pedometer:** to monitor the PA during waking hours and upload the data to a specific website.  **Incentives:** group and individual incentives including prizes and cash.  **We-Chat group:** created by each group captain to share daily steps number, communicate and motivate the participants to achieve the corresponding goals.  **CG:** required to complete the measures and no intervention applied.  **Underlying theory:** Social cognitive theory (SCT) | 1. Research team 2. WeChat group captain who motivates participants to achieve their own goals in the WeChat group. | PA and health-related outcome | **Primary outcome:**   1. **Physical activity:** measured by Chinese version of the short International Physical Activity Questionnaire (IPAQ) which includes 3 specific PA (VPA=vigorous PA, MPA= moderate PA, walking and sedentary time) 2. **Health related outcome:**  - **Height (cm):** self-reported - **Weight (kg) and Body Fat % (BF%):** measured by body fat meter - **Systolic BP and Diastolic BP:** measured by an electronic sphygmomanometer - **Waist circumference (WC) and Hip circumference (HC):** measured by tape manually - **BMI**   **Secondary outcome:**   1. **Job demand and control:** measured based on Karasek’s Job Content Questionnaire (JCQ) with 11 Likert-scale items (score 1-5, with 1 fully disagree, 5 fully agree) | **Significance difference at baseline between IG and CG:**   1. **WC (p=0.020):** IG= 85.1 ±9.3 cm; CG= 82.3±9.8 cm 2. **HC (p<0.001):** IG= 97.7 ±5.9 cm; CG= 94.8 ±6.2 cm 3. **BMI (p=0.005):** IG=23.7±3.0 cm; CG= 22.7±3.1 cm   **At post intervention, walking increased about 22% compared with baseline VPA**  **Significant changes in PA at post intervention:**   1. **Increased VPA in IG (p=0.048):** +394.5 ±774.8 METs 2. **Decreased MPA in both IG (p<0.01) and CG (p<0.01):** -66.4 ±137.9 METs 3. **Increased walking in IG (p<0.01):** +1164.7 ±894.3 METs   **Significant changes in health-related outcomes at post intervention:**   1. **Decreased SBP in IG (p=0.005):** -122.3 ±18.4 mmHg 2. **Increased DBP in CG (p=0.011):** +78.0 ±10.8 mmHg 3. **Decreased WC in IG (p<0.01):** -82.3 ±9.8 cm 4. **Decreased HC in IG (p<0.01):** -94.8 ±6.2 cm 5. **Decreased BF in IG (p<0.01):** 25.0 ±4.6% 6. **Decreased BMI in IG (p<0.01):** -22.7 ±3.1 kg/m^2^   **Significant association between:**   1. **Gender and WHR (p<0.001):** females showed larger decreased WHR 2. **Age and BF (p=0025):** age increased; BF decreased 3. **Age (p<0.027) and difference in METs for VPA (p<0.001) with BF**: older and higher difference showed larger decreased BF 4. **VPA and BMI (p=0.013):** higher difference, larger decreased BMI | Integrated group-based intervention programs contributed to comprehensive improvement in health-related outcomes.  Long-term evaluation is required to examine the potential of such an integrated intervention to promote PA. |
| Pillay (2014) | South Africa  Staff members of the Faculty of Health Sciences, University of Cape Town | Pilot study  10-week intervention with 2-week follow-up | **Voluntary**  **Baseline**  **Total (n)=**25  **Completed measure (n)=** 22  **Follow-up**  **Total (n)=** 19  **Gender=** 84.21% females  **IG**  **Total (n)= 11**  **Age=** 37.6±8.6 years  **Gender**= 81.8% females  **BMI=** 31.1±6.6 kg/m2 (Obese)  **CG**  **Total (n)=8**  **Age=**38.3±7.7 years  **Gender**= 78.5% females  **BMI=** 26.0±4.5 kg/m2 (Overweight) | **Inclusion criteria:**   1. Willing employees aged 21-49   **Exclusion criteria**:   1. Pregnant 2. Diagnosis or treatment of cancer 3. Any other physical/ clinical condition that made PA difficult 4. Contract workers whose employment end before 12-week follow-up measurement 5. Non-compliance to a min of 3 days of blinded pedometer wear at baseline | **IG:** Received biweekly individualised, emailed feedback (general supportive/ motivational messages and strategies to increase PA) based on electronic receipt of pedometer data.  **CG**: Received general motivational message biweekly but without pedometer feedback  **Underlying theory:** Transtheoretical model of behavioural change | Research team | Perception of the intervention and PA level | **Primary outcome:**   1. **Participants’ perceptions:** false, appeal, support and benefits of the intervention assessed through questionnaire during follow-up period.   **Secondary outcome:**   1. **PA level= measured at baseline and follow-up**  - **Steps per day:** recorded by pedometer and being classified as aerobic or non-aerobic according to Omron classification that integrates both intensity and duration. - Biometric and clinical measure: - **Waist circumferences (cm):** measured by tape measure - **Body fat:** measured by Omron body composition monitor (BF500) - **BMI:** weight (kg) divided by height (kg) - **Body weight (kg):**  measured by electric scale (Beurer PS 06) - **SBP and DBP:** measured by sphygmomanometer | **IG at 2-week follow up period after intervention:**   1. Average daily aerobic steps: decreased (-54±2746 steps) 2. Daily aerobic time (min): Increased (+0.9±23.0) 3. Daily steps: increased (+996±1748 steps)   **CG at 2-week follow up period after intervention:**   1. Daily steps: increased (+97±750 steps) | This pilot study provides useful information on the potential for PA improvements through pedometer in an employed, adult group.  In so doing, the study provides a basis to further pedometer-based interventions that can be applied in other contexts and settings and on a larger scale. |

1. **Job Performance (n=3)**

| **Author, Year** | **Study settings** | **Design /Duration** | **Population characteristics** | **Recruiting inclusion criteria** | **Mode of delivery / Underlying theory**  **IG:** Intervention Group  **CG:** Control or Comparison Group | **Intervention providers** | **Health behaviour targeted** | **Outcomes and measures** | **Result (Qual & Quant)** | **Main findings** |
| --- | --- | --- | --- | --- | --- | --- | --- | --- | --- | --- |
| Aliakbari (2020) | Iran  General dentists and dental specialists in Bojnourd city. | Quasi-experimental study  3-month intervention without follow-up | **IG**  **Total (n)=** 32  **Age=** 41.9±8.9 years  **Weight=** 69.3±13.4kg  **Gender=** 65.6% males  **Marital status=** 90.6% married  **Education=** 81.2% general  **Daily working hours=** 6.52±2.3 hours  **History of skeletal disease=** 51.1% No  **Exercise=** 62.5% No  **CG**  **Total (n)=** 31  **Age=** 37.6±8.5 years  **Weight=** 71.3±13.2 kg  **Gender=** 64.5% males  **Marital status=** 83.9% married  **Education=** 80.6% general  **Daily working hours=** 7±1.96 hours  **History of skeletal disease=** 48.9% No  **Exercise=** 64.5% No | **Inclusion criteria:**   1. Willing to participate 2. Lack of congenital skeletal problem 3. Able to use mobile phones and computers 4. Absence of corrective physiotherapy | **IG:** educational intervention developed based on predictive constructs using modern media.   - **Sending messages:** 1-2/day for a month to participants about changing behaviour as positive subjective norm group - **“Ergonomics” Telegram group:** participants to share knowledge to help each other improve musculoskeletal conditions - **Software package:** consisting of several applications was installed on participants’’ computers. - **Education:** Articles and trainings for posture and skeletal problems - **A Persian website:** designed based on participants’ needs   **CG:** received software package consisting of several applications, articles and corrective trainings as IG but not involved in Telegram group.  **Underlying theory:** Theory of Planned Behaviour | Research team | Effectiveness of the intervention, health and ergonomic conditions | **Primary outcome:** evaluated by a questionnaire that consisted of 3 parts   1. **Health conditions (Part 1):**  - **Personal info, daily activities and exercise and so forth**  1. **Knowledge and constructs of behavioural intention model (Part 2):** skeletal problems and ergonomic conditions, intention construct, attitude construct, subjective norms, perceived control construct with 5-point Likert scale (5= strongly agree, 1= strongly disagree) 2. **Ergonomics condition (Part 3)**  - **Nordic questionnaire**: evaluate musculoskeletal disorders - **Rapid Upper Limb Assessment (RULA):** evaluate ergonomic posture of dental practitioner by taking images of participants during their work and recorded the most repeated position, higher score indicates greater musculoskeletal pressure | **Significant difference of mean scores of constructs between IG and CG at pre-intervention:**   1. Perceived control (p=0.04）   **Significant difference of mean scores of constructs in IG at post-intervention:**   1. Attitude score (p=0.03)   Subjective norms, perceived control, attitude and behavioural intention had the highest predictive power in improving the health and ergonomic position of dentists, respectively. | Training based on the TBP using digital media was effective in the knowledge and attitude of ergonomic conditions of the dentists. However, it was not effective for behavioural intention. |
| Guo  (2020) | China  Clinical nurse | RCT  6-month intervention | **Voluntary**  **Baseline**  **Total (n)=** 197  **Eligible (n)=**102  **Included (n)=** 73 (with 71.57 adherence)  **IG**  **Total (n)=** 49  **Completer (n)=**33  **Age=** 27.82±5.42 years  **Gender=** 100% females  **Education=**90.9% with bachelor’s degree  **Burnout**= 2.29 ±0.51  **CG**  **Total (n)=** 53  **Completer (n)=** 40  **Age=** 28.73±5.10 years  **Gender=** 97.5% females  **Education=** (85.0% with bachelor’s degree  **Burnout=** 2.37 ±0.71 | **Inclusion criteria:**   1. Registered nurses from a Chinese tertiary general hospital 2. Working full-time and provided direct clinical care to patients 3. With Maslach Burnout Inventory-General Survey (MBI-GS) score > 1.5   **Exclusion criteria:**   1. Nurses had participated in the positive psychotherapies | **IG:** WeChat-based 3GT-positive psychotherapy   1. **WeChat circle:** to record three good things that were impressive each day and answer 2 questions: “Why did these good things happen?” and “What was your role in bringing them about?” 2. **Reminder messages for recording 3 good things**: sent to all the nurses at 8 pm by the researcher to remind them to increase the adherence of 3GT   **CG:** No intervention received  **Underlying theory:** Self Efficacy | Research team | Job performance, self-efficacy, burnout | **Primary outcome:** job performance consisting of three subscales namely job contribution, task performance and interpersonal support, measured by a 16-item scale with 6-point Likert  **Secondary outcome**: Self-efficacy was measured by the 10-item General Self-efficacy Scale with 4-point Likert scale  **Tertiary outcome**: Burnout was measured by the 16-item MBI-GS with three subscales including emotional exhaustion, cynicism and reduced professional efficacy using 7-point Linkert scale  **Baseline questionnaire:** socio-demographic info including age, gender, working department and length, education background, marital status and child, income/month (after tax), child/children, shift work | **Significant main intervention effect in both IG and CG**:   1. Job contribution (F= 6.43, p=0.01) 2. Task performance (F= 29.25, p=0.00) 3. Interpersonal support (F= 17.68, p= 0.00) 4. Self-efficacy (F= 5.06, p= 0.03)   **With higher scores in nurses in IG**  **Significant interactions between intervention and time**:   1. Job contribution (F= 8.379, p= 0.00) 2. Task performance (F= 19.501, p=0.00) 3. Interpersonal support (F= 11.009, p= 0.001) 4. Self-efficacy (F= 16.895, p= 0.00)   **Significant main time effects on self-efficacy (p=0.00)**  **Significant difference:**   1. Post-intervention scores for job performance (p <0 .05) and self-efficacy (p <0 .05) between IG and CG. 2. Scores for job performance and self-efficacy of the IG before (p<0.05) and after (p<0.05). | The WeChat-based 3GT positive psychotherapy on job performance and self-efficacy of nurses who suffered burnout was effective.  3GT focuses on enhancing nurses' positive perception of their personal strengths and favourable environments. These are critical for the promotion of job performance and self-efficacy. |
| Sasaki  (2021) | Vietnam,  Nurses from public tertiary hospital at national level | Three-Arm RCT  7-month intervention with  Follow-up at 7th month | **Voluntary**  **Baseline**  **Total (n)**= 1269  **Completed survey (n)**= 962 (75.82%)  **Meet criteria (n)=** 951, randomly allocated with 317 in each group (3 groups)  **Gender=** 84.9% females  **Education=** 46.7% vocational school  **Marital status=**83.6% married  **Employment=** 53.2% permanent  **Mean age=** 33.1 years (range 22 to 58). | **Inclusion criteria**:   1. Being employed full time as a registered nurse 2. Having internet access via a mobile device such as a smartphone.   **Exclusion criteria**:   1. Plans to change or leave the job in the next 7 months. 2. Being an assistant nurse and helper 3. Being temporary or part-time employed   **Inclusion criteria removed before the start of baseline survey**:   1. Having taken leave for >15 days for a physical or mental condition in the past 3 months 2. Undergoing treatment for a mental health problem from a mental health professional | **IG:** Two smartphone-based stress management programs were developed in the ABC Stress Management app.  **Program A:** 6-module CBT program with free-choice multi module program (complete 1 module/week in any order)  **Program B**: 6-module CBT program with fixed-sequential order multi module (complete 1 module/week in a fixed order)  **Program A & B**   1. Incompleter will receive weekly reminder messages. 2. Informal group chat (via social media apps such as Vober, Zalo, FB messenger) with researchers and hospital head nurses to deliver intensive technical support   **CG**:   1. Received intervention after the 7-month intervention period 2. Free to use any other mental health services as usual treatment.   **No theory applied** | The researchers developed the program based on discussions with Vietnamese nurses to consider the cultures and specific stressors that they could have at work. | Work engagement | **Secondary outcome**: Work engagement as the secondary outcome in the current study.   1. **Work engagement:** measured by short form of Utrecht Work Engagement Scale-9 item (UWES-9) which consists of 3 sub-scales (vigor, dedication, adsorption) with self-report 7-point rating scale (0=never, 7=every day) 2. **Demographic:** gender, age, education, marital status, employment contract 3. **Intervention effectiveness:** measured immediate effect at 3-month follow-up and longer-term effect at 7-month follow-up through paper-based self-administered survey questionnaire   **Primary outcome**: Anxiety and depressive symptoms in the previous study, assessed by Depression, Anxiety, and Stress Scale–21-item [DASS-21] | **Work engagement scores in both IG:**   1. Increased from baseline to 3-month follow-up but decreased at the 7-month follow-up 2. **At 3-month follow-up:**  - Program B showed significant improvement (P<0.05) with a small effect size. - Program A showed non-significant improvement (P=0.07)  1. **7-month follow up:** neither program achieved effectiveness.   **Work engagement scores in both CG:**   1. Steadily increased from baseline to 7-month follow-up | A fixed order (program B) delivery of a smartphone-based stress management program was effective in improving work engagement in nurses in Vietnam. However, the effect was small and only temporary.  Further improvement of this program is required to achieve a greater effect size and more sustained, longer lasting impact on work engagement. |

1. **Stress (n=3)**

| **Author, Year** | **Study settings** | **Design /Duration** | **Population characteristics** | **Recruiting inclusion criteria** | **Mode of delivery / Underlying theory**  **IG:** Intervention Group  **CG:** Control or Comparison Group | **Intervention providers** | **Health behaviour targeted** | **Outcomes and measures** | **Result (Qual & Quant)** | **Main findings** |
| --- | --- | --- | --- | --- | --- | --- | --- | --- | --- | --- |
| Dincer and Inangil (2021) | Turkey  Nurses caring for CVID-19 patients in a university hospital | RCT  A 20-mins intervention for each nurse without follow-up | **Baseline**  **Met criteria (n)=** 80  **Completed study (n)=** 72  **IG**  **Total (n)=** 35  **Age=** 33.45±9.63 years  **Gender=** 91.4% females  **Marital status=** 60% married  **Education=** 62.9% with bachelor’s degree  **CG**  **Total (n)=** 37  **Age=** 33.379.58 years  **Gender=** 86.5% females  **Marital status=** 59.5% married  **Education=** 70.3% with bachelor’s degree | **Inclusion criteria:**   1. Not having any psychiatric diagnoses 2. Not taking any courses about coping with anxiety and stress   Voluntary participation | **IG:** received a 20-mins guided online group Emotional Freedom Techniques (EFT) treatment by showing the participants a picture of the acupressure points and ways to tap.  **CG:** no EFT treatment received between the completion of 2 SUD burnout tests.  **No theory applied** | **The research team:** EFT treatment was provided by the first author, who was certified in EFT | Stress, anxiety and burnout level | **Primary outcome:**   1. **Stress levels:** measured by subjective units of distress scale (SUD) based on 0-10 scale (0= no distress, 10= distress unbearable) 2. **Anxiety levels:** measure the State Anxiety Scale which consists of 20 questions about emotions, thoughts and behaviours related to anxiety based on scale of 0 to 4 (0=no anxiety, 4= extreme anxiety) 3. **Burnout levels:** measured by a 21-item Burnout Scale with 7-point Likert type scale. (1= never, 7=always) | **Significant difference in IG at post intervention** (p<.001)**:**   1. **Stress levels:** Reduced mean SUD score 2. **Anxiety levels** Reduced anxiety score 3. **Burnout levels:** Reduced burnout score   **CG showed no statistically significant changes on these measures (p>0.05)** | A single online group EFT session reduced stress, anxiety, and burnout levels in nurses treating COVID-19. |
| Divya (2021) | India  Healthcare providers (HCPs) | A pilot study with a single arm pre-post design  4-day intervention workshop with follow-up at 40 days | **Baseline**  **Included (n)=** 100  **Completed survey (n)=** 92  **Age=** 43.1±11.1 years  **Gender=** 58.7% females  **Medical practice=** 17.4±10.7 years  **Smoking:** 86.9% No  **Alcohol use:** 67.4% No  **Exposed to COVID-19=** 52.2% No  **Treated COVID-19 patient=** 69.6% No  **Follow-up**  **Completed assessment (n)=** 32  **SKY practice**  **Daily=** 46.7%  **4-5 times/day=** 20%  **2-3 days/week=** 13.3%  **At least once/week=** 20% | **Inclusion criteria:**   1. HCPs including doctor, podiatrist, dentist, chiropractor, clinical psychologist, optometrist, nurse practitioner, nurse midwife, clinical social worker who is authorised to practice by the state 2. The organisers of 45 online SKY workshops which involved 7597 participants. | **IG:** received a 4-day online breath and meditation workshop, Sudarshan Kriya Yoga (SKY) delivered by trained instructors with a 2-hour session/ day through video conference. Participants also learnt the 35-min home practice, including Pranayama, Bhastrika and SKY breathing to be practised at home daily.  **No CG and theory applied.** | Experienced SKY instructors trained by the Art of Living Foundation facilitated the workshops. | Wellbeing of HCPs (Depression & Anxiety, Resilience, Life satisfaction and Sleep quality) | **Primary outcome:**   1. **Depression and Anxiety:** measured by a set of three 7-item self-reported Depression, Anxiety and Stress Scale (DASS-21) based on scale of 0-3. 2. **Sleep Quality:** measured by Pittsburgh Sleep Quality Index (PSQI) which has 19 questions assessing 7 components of sleep including quality, latency, duration, habitual sleep efficiency, sleep disturbances, the use of sleep medication and daytime dysfunction using 3 Likert scale. 3. **Resilience:** measured by a 25-item self-rated Connor-Davidson Resilience Scale with 4 Likert, higher score reflects greater resilience. 4. **Life satisfaction:** measured by a 5-item Satisfaction with Life Scale regarding global cognitive judgements of life satisfaction with 7 Likert, higher scores reflect great satisfaction. | **Significant difference in the scale scores:**   1. **Depression, anxiety, and stress:** reduced scores reported for all the three states at post-intervention (p<0.001) 2. **Resilience:** Increased resilience at post-intervention (p<0.001) and greater increase at follow-up phase (p=0.015). 3. **Life satisfaction:** increased life satisfaction at post-intervention (p<0.001) and greater increase at follow-up phase (p< 0.001). 4. **Quality of sleep:** reduced scores immediately after the program (p<0.001). The % population (PSQI <5 score): +54.3% at post-intervention, +68.75% in follow-up phase. | SKY breathing technique had a positive impact on the well-being of healthcare professionals during the pandemic. Participants experienced improved quality of sleep, enhanced satisfaction with life, and increased resilience after SKY. |
| Pendse (2012) | India  Service sector employees | Pilot study with part 1 (P1) survey and part 2 (P2) intervention  10-working day intervention without follow-up | **Voluntary**  **P1 included (n)=** 81  **Age=** 25-50 years  **P2 included (n)=** 10  **Age=** 25-35 years  **Gender=** 60% females  **IG (n)=** 5  **CG (n)=** 5 | **Inclusion criteria for P2 intervention:**   1. Employees in P1 high on Smith Stress Symptoms Inventory (SSSI) domain of Worry/Negative Emotion (>60%) | **IG:** receive two 20-40 seconds stimuli which in the forms of pictures, videos and text through official emails every day for 10 working days. The stimuli were designed based on 5 basic themes namely, Expressions, Humour (jokes), Nature, Colours and India which could induce positive emotions of Joy, Serenity, Interest, Pride and Belongingness.  **CG:** Did not receive intervention  **Underlying theory:** Influential theory | Research team | Quality of Work Life (P1) and mental well-being (P2) | **Questionnaire in P1:** assess quality of work life   1. **Career and job satisfaction perceived absence of work stress:** measured by 15-item Oxford Happiness Questionnaire, 51-item Abhyankar Quality of Work Life Scale, 25-item Connor-Davidson Resilience Scale   **Questionnaire in P2:** assess Affect balance and negative emotion/ worry   1. Affect Balance= 10 questions Bradbrun’s affect Balance scale 2. Emotion/worry= Smith Stress Symptoms Inventory | **Happiness positively and significantly related to**   1. Quality of Work Life (R=0.378, P<0.01) 2. Resilience (R=0.365, P<0.05)   CG on affect balance significantly lower than the gain scores of IG (U=14, P<0.05) | Web-based interventions can enhance employee’s happiness but there’s limitation on small sample size. |

1. **Sleep (n=2)**

| **Author, Year** | **Study settings** | **Design /Duration** | **Population characteristics** | **Recruiting inclusion criteria** | **Mode of delivery / Underlying theory**  **IG:** Intervention Group  **CG:** Control or Comparison Group | **Intervention providers** | **Health behaviour targeted** | **Outcomes and measures** | **Result (Qual & Quant)** | **Main findings** |
| --- | --- | --- | --- | --- | --- | --- | --- | --- | --- | --- |
| Montagni (2019) | China, France, Spain, UK  Employees of eight company sites in four countries | Pilot study  Two phase (T0 and T1) intervention of 5 days each.  Follow-up (T1) after 6 months | **Voluntary**  **First phase (T0))**  **Total (n)=** 834  **Second phase (T1)**  **Total (n)=** 291 (34.9% follow-up rate)  **Age=** 36.1% with 18-34 years  **Gender=** 56.7% females | **Inclusion criteria:**   1. Aged 18-64 2. With permanent contract, fixed-term contract, apprenticeship contract, temporary employees, service providers and trainees. 3. Voluntary participation | **IG:** asked to use WarmUapp tablet application which consist of 27 screens (23 screens for questions,  3 screens for partial survey answers,1 screen for survey results and personalised recommendations to improve sleep quality)  Blending of face-to face meeting and tablet application are involved in both T0 and T1 phase.  **No CG and theory applied** | A local team (managers, HR, communication and IT officers) was built to in charge of adapting and implementing the pilot intervention and was instructed by HQ WarmUapp^TM^ committee | Sleep awareness | **Primary outcome**:   1. **Change in sleep status which measured**  - **Total sleep duration:** <5hrs, ≥ 5hrs and < 7hrs, ≥ 7hrs - **Sleep efficiency:** <85%, ≥85% and <90%, ≥ 90% - **Sleep debt:** <90mins, ≥90mins and <120mins, ≥120mins - **Insomnia:** no sleep difficulties, sleep difficulties, insomnia - **Sleepiness:** <10, ≥10 and <16, ≥16  1. **WarnUapp^TM^ effectiveness:** measured user satisfaction, feedbacks and ideas through structured interview composed of 3 open-ended questions | **Significant difference in sleep status at post intervention (follow-up phase):**   1. **Increased total sleep duration (p=0.046)**: 1.2 times more likely to sleep ≥ 7hours than between 5-7hours 2. **Decreased sleep debt (p=0.019):** 2.8 times less likely to present severe sleep debt (≥7hours) 3. **Decreased sleep difficulties between two phases (p<0.001):** 2.5 times lesser 4. **Decreased sleepiness (p=0.026):** 1.7 times lesser 5. **Sleep problem:** females 2 times more likely to suffer in both phases (p=0.006)   **Effectiveness of WarnUapp^TM^:** All interviewees were satisfied of the intervention | Interventions blending face-to-face and web-based approaches show  promise for effective promotion of sleep awareness at the workplace. |
| Nourian (2021) | Iran  Nurses working in 2 COVID-19 care wards in one selected hospital. | RCT  7-week intervention | **Voluntary**  **Baseline**  **Total (n)=** 44  **Included (n)=** 41 (93.18% completion rate)  **Age=** 35.60 ± 8.21 years  **Gender=** 59.6% females  **Education=** Bachelor’s degree  **Work experience=** 7.97 ± 5.55 years.  **Both IG & CG homogenous:**  Gender (p= 0.23)  Age (p= 0.33)  Initial sleep quality score (p=0 .86) | **Inclusion criteria**   1. Obtains at least a bachelor’s degree in nursing 2. Working in the COVID-19 care ward for a max of 2 weeks before the start of the study 3. Willing to participate in research 4. Not having performed mindfulness exercises previously 5. Access to mobile phones 6. Nonuse of drugs related to psychological disorders at the time of the study.   **Exclusion criteria:**   1. In charge the ward voluntarily or by nursing management for any reason at the time of the study 2. Desire to leave the study or develop COVID-19 disease and taking a absence leave 3. Fail to do the exercises > a week according to the participant’s statement 4. Fail to complete/not complete all items in the questionnaires. | **IG**: Mindfulness-based stress reduction (MBSR) training program via WhatsApp group.   1. Completed 2 questionnaires on Porsline website 2. MBSR training program for 7 weeks with  - **Training media:** Content included audio files of meditation, video files of yoga exercise, reading files about the nature of mindfulness and its benefits, several audio and video files containing speeches delivered by professionals related to mind exercises that were translated, dubbed, or subtitled in Persian. - A logbook to write down experiences and meditating on the exercise   **CG:** completed 2 questionnaires on Porsline website, received music and training file without WA application.  **No theory applied** | Training media prepared by researchers in collaboration with a group of nursing students | Sleep quality, mindfulness | **Personal information questionnaire:** age, gender, education, nursing experience, marital status, having children, shift conditions, number of nursing hours week, history of sleep disorders o previous psychological disorders  **Pittsburgh Sleep Quality Index (PSQI) tool**: subjective sleep quality, sleep latency, sleep duration, habitual sleep efficiency, sleep disturbances, use of sleep medication and daytime drowsiness.  *(Overall score ≥ 5= poor sleep quality) | **Significant different between Subscales scores before and after the intervention in IG showed the effectiveness of the MBSR program on**:   1. Subjective sleep quality (p=0.002) 2. Sleep latency (p=0.039) 3. Habitual sleep efficiency (p=0.046)   **Significant increase after 8 weeks in IG:**   1. Subjective sleep quality (p=0.020) 2. Daytime drowsiness (p=0.007) 3. Total sleep quality score (p=0.001).   **Significant differences between IG and CG before and after the intervention**   1. Sleep latency (p=0.020) 2. Subjective sleep quality (p=0.000)   **Satisfaction with WarnUapp^TM^** : All interviewees were satisfied of the intervention | The results showed that total sleep quality did not change among the participants in the IG before and after the intervention, but it increased significantly in the CG. The MBSR program may be effective in improving the sleep quality of nurses. |

**Near Missed Paper**

| **Author, Year** | **Study settings** | **Design /Duration** | **Population characteristics** | **Recruiting inclusion criteria** | **Mode of delivery / Underlying theory**  **IG:** Intervention Group  **CG:** Control or Comparison Group | **Intervention providers** | **Health behaviour targeted** | **Outcomes and measures** | **Result (Qual & Quant)** | **Main findings** |
| --- | --- | --- | --- | --- | --- | --- | --- | --- | --- | --- |
| Wasemann (2016) | Afghanistan  Soldiers from medical military unit to be deployed abroad | RCT  6-month intervention with 2-week follow up after the deployment | **Voluntary**  **Baseline**  **Total (n)=** 73  **Included (n)=** 67  **Age=** 28.46 ± 5.42 years  **Gender=** 47 males (70%)  **Deployment=** 55.22% never deploy abroad  **Working department=** 68.7% non-commissioned officers  **Education=** 58.2% with intermediate school education level  **Marital status=** 67.2% with partners  **IG (n) 36**  **CG (n)= 31** | **Inclusion criteria:**   1. Members of military units to be deployed abroad.   **Exclusion criteria:**   1. With diagnosis of an acute mental disorder according to Internal Classification of Diseases. | **IG:** received a 1.5-day CHARLY training, a computer-assisted method and biofeedback focused on reducing PTSD and current mental state.  **CG:** received 1,5-day training by a military psychologist (conventional way), comprising the exactly the same psychoeducation.  **No theory applied.** | The research team | **Mental health (PTSD)** | **Outcome:**   1. **Current mental state:** measured the psychological stress using the 53-items on a 5-point Likert scale Global Severity Index (GSI) of the Brief Symptom Inventory (BSI) 2. **Attitudes and knowledge:** deployment-related stress, traumatic stress and PTSD was measured by a 16-item questionnaire with 5-point scale and 15-item MCQ questionnaire. 3. **Critical incidents on Foreign Deployment:** assessed by German version of the Combat Experiences Scale of the MHAT of the US armed forces. 4. **PTSD**: measured by PDS, a self-evaluated German version of the Post-traumatic Stress Diagnostic Scale | **Attitude change:**   1. Overall significant difference between t1, t2 and t3 measurement (*p* < 0.001) with less prejudiced, more realistic and healthier. 2. Significant positive effect in IG (p=0.045)   **Gain in knowledge:**   1. Significant increase in overall group throughout t1, t2 and t3 measurements (p<0.001) 2. Significant increase in between IG (p<0.001)   **Mental State:**   1. Significant deterioration in overall group (p=0.003) 2. Significant deterioration in CG (p=0.033)   **PTSD**   1. Significant difference between IG and CG in global impairment (p=0.028) 2. Significant different in three PDS subscales: re-experiencing (p=0.02) , avoidance (p=0.039) and arousal (p=0.031) | The study results tentatively indicate that highly standardised, computer-based primary prevention of mental disorders in soldiers on deployment might be superior to other more personal and less standardised forms of prevention. |
