# Supplementary material for: A scoping review of digital workplace wellness interventions in low- and middle-income countries
Source: PLoS One. 2023 Feb 28;18(2):e0282118. doi: 10.1371/journal.pone.0282118 (PMC9974126; doi:10.1371/journal.pone.0282118)
Supplement: Attachment — Submitted filename: ReviewerComments_10.03.22.docx [file pone.0282118.s004.docx]

**Overall, this article provides important and much-needed information on employer-sponsored digital workplace interventions in low and middle-income countries (LMICs). I applaud the authors for addressing this gap and conducting a thoughtful and rigorous scoping review of the literature. However, I have several major concerns regarding the Methods and Results. I believe addressing these concerns will make for a more informative, actionable, and relevant article.**

**Major:**

1) The stated goal of the paper was to provide a comprehensive synthesis of current evidence in relation to the effectiveness, feasibility, and acceptability of the digital workplace wellness intervention in the LMICs. However only very basic information is provided on the effectiveness, feasibility, and acceptability of programs. The Results section instead focuses on intervention and design components/qualities rather than findings related to their effectiveness, feasibility, and acceptability. Some of the latter elements are discussed in the third paragraph of the Discussion, but the main text Results do not adequately report these findings at a summary level. Information on effectiveness in the main article is largely in the form of a “conclusion” column in Table 2. I have two recommendations to address this concern:

-The Results section would benefit greatly from three high-level summary sections on effectiveness, feasibility, and acceptability (e.g., how well interventions worked, which health conditions or behaviors saw the most benefit, which intervention design types realized the most benefits, if information on effectiveness/feasibility/acceptability was mixed or lacking, etc.). I see very little reporting on the feasibility and acceptability of interventions. If it is absent in the underlying studies, please state this.

-S3 provides more informative results on effectiveness than Table 2. I recommend that the authors distill some of the information on statistical significance from S3 into a simplified column in Table 2, at least for the quantitative studies (which comprised all but one of the articles). Presenting the information in a simplified but study-specific column in Table 2 would help overcome the authors’ concern that it is difficult to report summary findings due to the difficulty in comparing or discerning “a clear pattern of effectiveness among the 24 studies.”

2) While the authors opine on the potential merits of digital-based interventions in LMICs, it seems that the limitations of such interventions in LMICs are overlooked. Please incorporate text into the Literature Review and Discussion sections that address this. For example, what are implications regarding generalizability of digital wellness interventions to certain areas of LMICs? Many areas of LMICs have poor access to broadband and electronic devices (e.g., home computers, internet, phone service, equipment cost, etc.). Additionally, I would recommend the authors add information about the size of the companies into their Results, and similarly, incorporate into the Literature Review any existing literature about the effectiveness of digital workplace interventions based on company size. One could imagine that tech-based wellness programs would be more cost-effective and successful for larger companies or those with greater fiscal infrastructure resources to implement the programs. The development and roll-out of digital interventions may require significant effort, finances, and resources that are not available to many employers in LMICs.
3) In general, the inclusion/exclusion criteria need to be more specific. Some of the information is presented in the Results, but the article would benefit from clarifying these inclusion/exclusion criteria in the Methods and in Table 1. For example, please address the following questions.

-It appears from the Results section (Study Characteristics) that pilot studies were included. Please clarify this in the Methods. This is important because the Table 1 exclusion criteria (“Protocols and study designs”) might imply that pilot studies were excluded, whereas the inclusion criteria of “design-related outcomes” and “feasibility” measures might imply that they were included.

-It appears from the Results section that interventions did not have to be employer-implemented (e.g., third party implementation such as that by an insurance company or government). Please include this information earlier on in the inclusion/exclusion criteria.

-Were there situations in which multiple distinct articles reported results from the same research study or wellness program and if so, how did you handle inclusion/exclusion of those articles?

-It appears from Table 2 that you included articles even if the wellness intervention was implemented for both LMICs and non-LMICs in the same sample. Please clarify this within Table 1 or somewhere in the main text, as well as whether results from the LMIC subsamples in those articles could be teased apart from the high-income countries, or in contrast, whether the pooled results were used.

-Students are listed as an excluded population in Table 1. However, Table 2 includes an article by Beleigoli (2020) that includes both students and university staff. Please clarify how this data was handled. Were you able to tease out data for staff from that of students?

**Minor:**

4) I gather from S2 that the years searched were from 2010-2021. Please add this information to the main text in either lines 90-91 or somewhere nearby. Is there a specific rationale for choosing these years?
5) Line 103 Did each of the two reviewers screen all articles? Or were articles divided among the two reviewers? Please provide more detail on the allocation of articles to reviewers in in lines 118-119 as well.
6) In Table 2, please define all abbreviations (e.g., RCT, w/o) in the footnotes for the reader. Do not define abbreviations within the table (e.g., (MBSR, SE, SD, etc.). In the main text, there are instances when the same acronym is defined more than once (e.g., RCT in Study Characteristics and Participant Characteristics). Please carefully review the use of acronyms throughout the manuscript.
7) In the Results, please describe how many interventions were purely digital versus how many incorporated parallel in-person components.
8) In Figure 3, the color-coded legend has a max N of N=27,406. Should this not be N=27,466 to reflect the number of participants from India?
9) In the Participant Characteristics of the Results section, it would be helpful to describe whether studies targeted at-risk populations versus general employees overall.
10) Future Research Directions: Please elaborate on what is meant by the statement that there was “no clear consensus on applying theoretical frameworks to the development of digital workplace wellness interventions in LMICs”. I was actually surprised that more than half of the articles were theoretically grounded. In my experience, this proportion parallels what we see in employer-sponsored wellness interventions based in high-income countries.
11) Though no formal review was conducted on the quality of underlying studies/research articles, a sentence or two on the perceived quality of studies would facilitate interpretation of the findings.
