# Supplementary material for: A scoping review of digital workplace wellness interventions in low- and middle-income countries
Source: PLoS One. 2023 Feb 28;18(2):e0282118. doi: 10.1371/journal.pone.0282118 (PMC9974126; doi:10.1371/journal.pone.0282118)
Supplement: Attachment — Submitted filename: The authors have sufficiently addressed all the reviewer concerns and comments.docx [file pone.0282118.s006.docx]

The authors have sufficiently addressed all the reviewer concerns and comments.
